# Severe Immune Thrombocytopenia Associated With Newly Diagnosed Rheumatoid Arthritis and Complicated by a Spontaneous Presacral Haematoma: A Case Report

**DOI:** 10.7759/cureus.116964

**Published:** 2026-09-26

**Authors:** Nyein Su Win, Wine Ohn Chit

**Affiliations:** 1 Accident and Emergency, Peterborough City Hospital, North West Anglia NHS Foundation Trust, Peterborough, GBR; 2 Acute Medicine, Aintree University Hospital, Liverpool University Hospitals NHS Foundation Trust, Liverpool, GBR

**Keywords:** immune thrombocytopenia, presacral haematoma, primary, rheumatoid arthritis, romiplostim, secondary

## Abstract

Immune thrombocytopenia (ITP) is an acquired autoimmune disorder characterised by isolated thrombocytopenia and a variable bleeding phenotype. Patients may be asymptomatic or may present with bleeding ranging from minor skin or mucosal manifestations to uncommon, potentially life-threatening internal haemorrhage. Its occurrence with rheumatoid arthritis (RA) is uncommon, and severe disease may require treatment beyond corticosteroids and intravenous immunoglobulin (IVIG). We report the case of a 64-year-old woman with recently diagnosed seropositive RA who was found to have a platelet count of 0 x 10^9^/L after presenting with a purpuric rash and recurrent epistaxis. Haemoglobin and white cell count were preserved, and investigations did not identify an infectious, malignant, or bone marrow cause. The platelet count remained critically low despite high-dose dexamethasone, two doses of intravenous immunoglobulin (IVIG), and platelet transfusions. Romiplostim was commenced on day 6, producing a progressive platelet response. Cross-sectional imaging undertaken during the admission identified a presacral haematoma with an initially suspected pseudoaneurysm; repeat triple-phase CT showed stability without active bleeding. The patient was discharged on weekly romiplostim, and her platelet count was 197 x 10^9^/L at the 65-day post-discharge follow-up assessment. This case highlights the rare association between RA and ITP, the importance of excluding alternative causes of thrombocytopenia, and the value of early thrombopoietin receptor agonist therapy when first-line rescue treatment is unsuccessful, as well as the rare occurrence of a pre-sacral hematoma with bleeding at an unusual anatomical site as a complication of severe thrombocytopenia.

## Introduction

Immune thrombocytopenia (ITP) is an acquired autoimmune disorder defined by a platelet count below 100 × 10⁹/L in the absence of another cause of thrombocytopenia [1,2]. Its pathophysiology involves autoantibody- and T-cell-mediated platelet destruction together with impaired megakaryocyte platelet production [3]. The incidence varies according to age and population; a nationwide French study reported an incidence of 2.9 per 100,000 person-years, with a higher incidence among people older than 60 years [4]. ITP is classified by duration as newly diagnosed (less than three months), persistent (3-12 months), or chronic (more than 12 months) [1].

The clinical phenotype ranges from asymptomatic thrombocytopenia to petechiae, purpura, mucosal bleeding, and uncommon but potentially life-threatening internal haemorrhage [2,4]. Bleeding risk is influenced by more than the platelet count alone; age, previous bleeding, comorbidity, and exposure to antiplatelet, anticoagulant, or non-steroidal anti-inflammatory drugs are also relevant. Nevertheless, very low platelet counts, particularly below 10-20 × 10⁹/L, are associated with an increased risk of bleeding [2].

ITP may be primary or secondary to infection, lymphoproliferative disease, immunodeficiency, medication exposure, or systemic autoimmune disease [2,3]. Its coexistence with rheumatoid arthritis (RA) has been reported only infrequently, and the available literature consists largely of small case series and individual reports with variable responses to glucocorticoids, intravenous immunoglobulin (IVIG), and other immunomodulatory treatment [5]. We present a case of severe newly diagnosed ITP in a woman with recently diagnosed seropositive RA, refractory to initial corticosteroid and IVIG therapy and complicated by a spontaneous presacral haematoma, with subsequent response to romiplostim.

## Case presentation

A 64-year-old woman with a recent diagnosis of seropositive RA was referred by her general practitioner to the acute medical team after routine blood testing showed a platelet count of 0 x 10^9^/L. Rheumatoid factor was 17 IU/mL, while anti-cyclic citrullinated peptide antibody was negative at 1.9 U/mL. Methotrexate had been planned but had not yet been commenced; her joint symptoms were being managed with meloxicam. She described a three-day history of a non-pruritic purpuric rash and two episodes of epistaxis. She reported no haematemesis, melaena, haematuria, menorrhagia, headache, focal neurological symptoms, or other bleeding. Her regular medication included lansoprazole, amitriptyline, atorvastatin, diazepam, gabapentin, and meloxicam. Her medical history also included cholecystectomy, spinal osteoarthritis, and a previous L1 compression fracture. She had a strong family history of RA in her father and mother.

On examination, she was afebrile and haemodynamically stable. There was no active nasal or gingival bleeding. A purpuric rash was visible, but no lymphadenopathy or hepatosplenomegaly was present. The presacral haematoma was clinically occult, and no localising symptoms suggesting pelvic bleeding were documented before imaging.

Investigations

Laboratory testing confirmed profound isolated thrombocytopenia. Haemoglobin, white cell count, renal function, and liver biochemistry were preserved (Table 1). Infectious serology was performed to exclude secondary causes and was negative. Stool microscopy, culture, and sensitivity were also negative. Serum protein electrophoresis showed polyclonal gammaglobulinaemia without a monoclonal band, and immunoglobulin G was within the reference range.

**Table 1:** Clinically relevant investigations at presentation and during the secondary-cause work-up eGFR: estimated glomerular filtration rate

Investigation	Patient value	Reference range
Platelet count (x10^9^/L)	0	150-400
Haemoglobin (g/L)	136	115-165
White cell count (x10^9^/L)	5.2	4.0-11.0
Prothrombin time (seconds)	10.5	9.4-12.4
Activated partial thromboplastin time (seconds)	24.1	24-36
International normalised ratio	0.94	0.80-1.25
Sodium (mmol/L)	136	133-146
Potassium (mmol/L)	4.1	3.5-5.3
Chloride (mmol/L)	105	95-108
Creatinine (micromol/L)	57	45-84
eGFR (mL/min/1.73m*2)	>90	>60
Total bilirubin (micromol/L)	13	0-21
Alanine aminotransferase (IU/L)	17	<33
Aspartate aminotransferase (IU/L)	40	10-50
Alkaline phosphatase (IU/L)	67	30-130
C-reactive protein (mg/L)	26	<5
Erythrocyte sedimentation rate (mm/hour)	107	0-17
Lactate dehydrogenase (IU/L)	211	<250
Antinuclear antibody screen	0.2	<0.7
Complement C3 (g/L)	1.46	0.75-1.65
Complement C4 (g/L)	0.12	0.14-0.54
Cardiolipin IgG antibody (GPL-U/mL)	9.9	<10 (negative)
Immunoglobulin G (g/L)	12.2	6.0-16.0
Serum protein electrophoresis	Polyclonal gammaglobulinaemia; no monoclonal band	No monoclonal band

Treatment and clinical course

Treatment was initiated with oral dexamethasone 40 mg daily, IVIG 45 g, and one unit of platelets. The platelet count remained critically low, as shown in Table 2. Following multidisciplinary discussion with Rheumatology, methotrexate was deferred. A second dose of IVIG was administered, dexamethasone was continued, and a further platelet transfusion was given when petechiae developed.

**Table 2:** Platelet-count response according to treatment phase Romiplostim was administered weekly and adjusted according to the platelet response. IVIG: intravenous immunoglobulin

Treatment phase	Platelet count (×10⁹/L)	Clinical response
Before treatment	0	At presentation
Dexamethasone, first IVIG dose, and platelet transfusion	0	Persistent severe thrombocytopenia
Second IVIG dose and further platelet transfusion	1	No meaningful initial response
Week 1 after romiplostim initiation	12	Early platelet response
Week 2 after romiplostim initiation; weekly outpatient treatment	48	Continued platelet response
Week 3 after romiplostim initiation; weekly outpatient treatment	119	Progressive platelet response
Continued weekly romiplostim with dose adjustment	131	Fluctuating platelet response
Continued weekly romiplostim	265	Platelet count within the reference range
Continued weekly romiplostim	357	Highest documented platelet count
Reported follow-up assessment during weekly romiplostim	197	Platelet response maintained at that assessment

Because of the severity of thrombocytopenia and the limited response to first-line rescue therapy, bone marrow examination was undertaken. It showed no alternative marrow abnormality and findings supportive of peripheral immune platelet destruction. Romiplostim 600 micrograms was commenced on day 6 together with regular tranexamic acid. Following romiplostim initiation, the platelet count began to rise, reaching 12 x 10^9^/L and subsequently continuing to recover.

Computed tomography (CT) of the neck, chest, abdomen, and pelvis, performed to investigate a possible secondary trigger, demonstrated a localised right presacral haematoma involving the bulky right piriformis muscle and extending anteriorly (Figures 1, 2). A focal 15 mm high-attenuation structure within the haematoma was reported as a possible sentinel clot or pseudoaneurysm arising from adjacent sacral or iliac vessels (Figure 3). The initial examination did not include an arterial phase. Vascular surgery advised interval imaging. A repeat triple-phase CT performed two days later demonstrated a grossly stable presacral haematoma without active contrast extravasation (Figure 4). She remained haemodynamically stable and had no further clinical bleeding.

**Figure 1:**
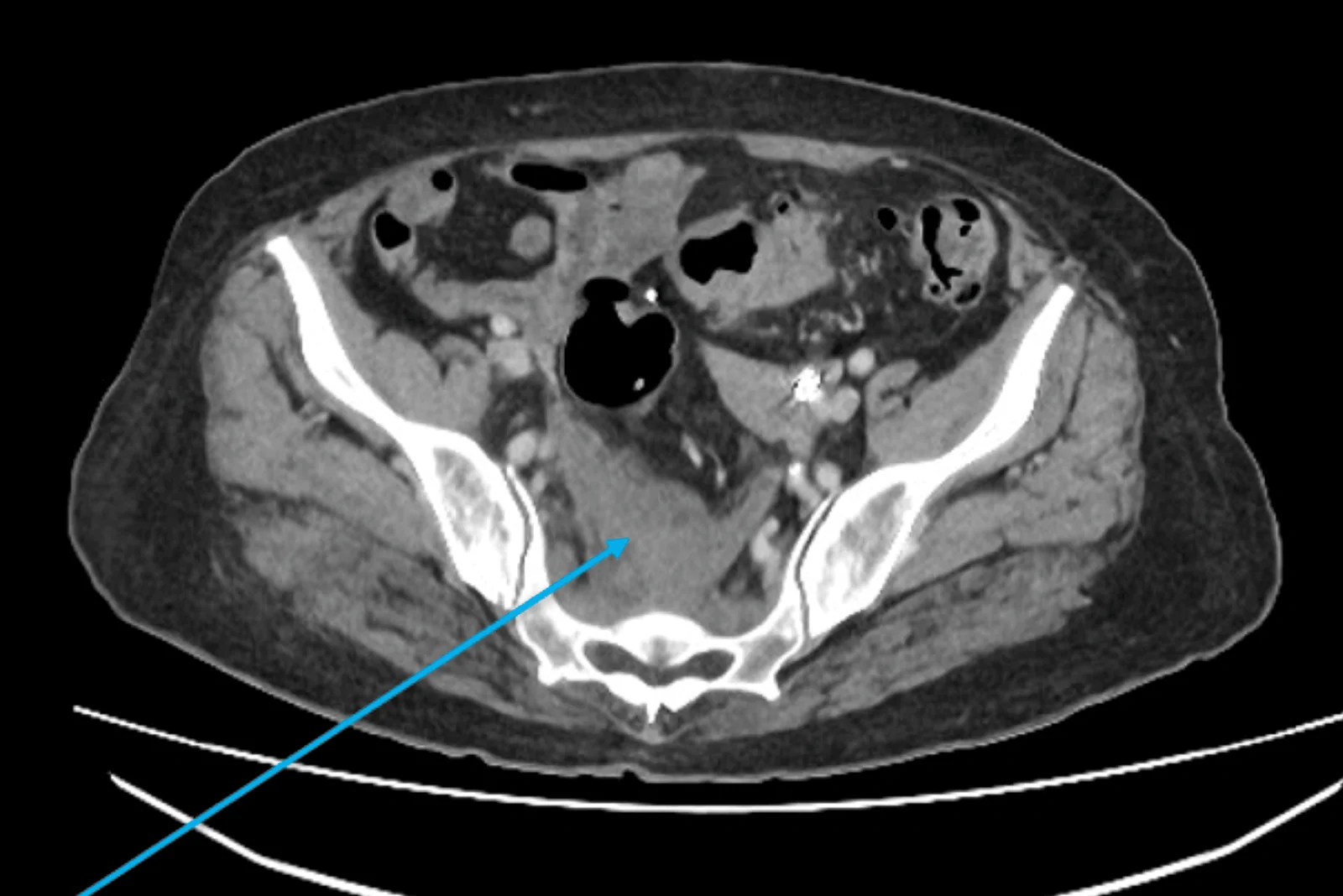
Initial contrast-enhanced axial CT of the pelvis demonstrating the right presacral soft-tissue-density haematoma (blue arrow), which involves the right piriformis muscle and extends anteriorly.

**Figure 2:**
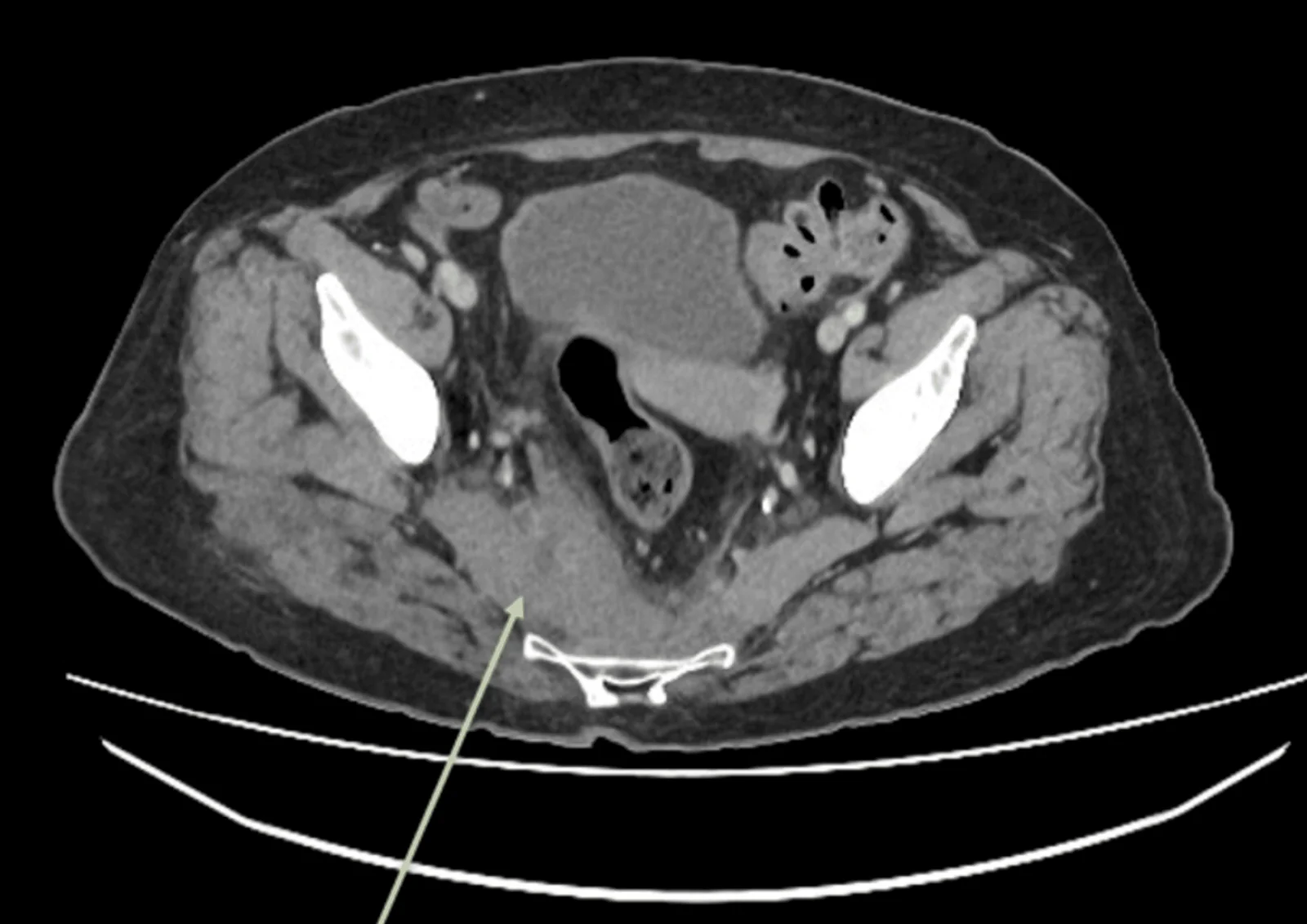
Initial contrast-enhanced axial CT of the pelvis demonstrating the bulky right piriformis component (arrow).

**Figure 3:**
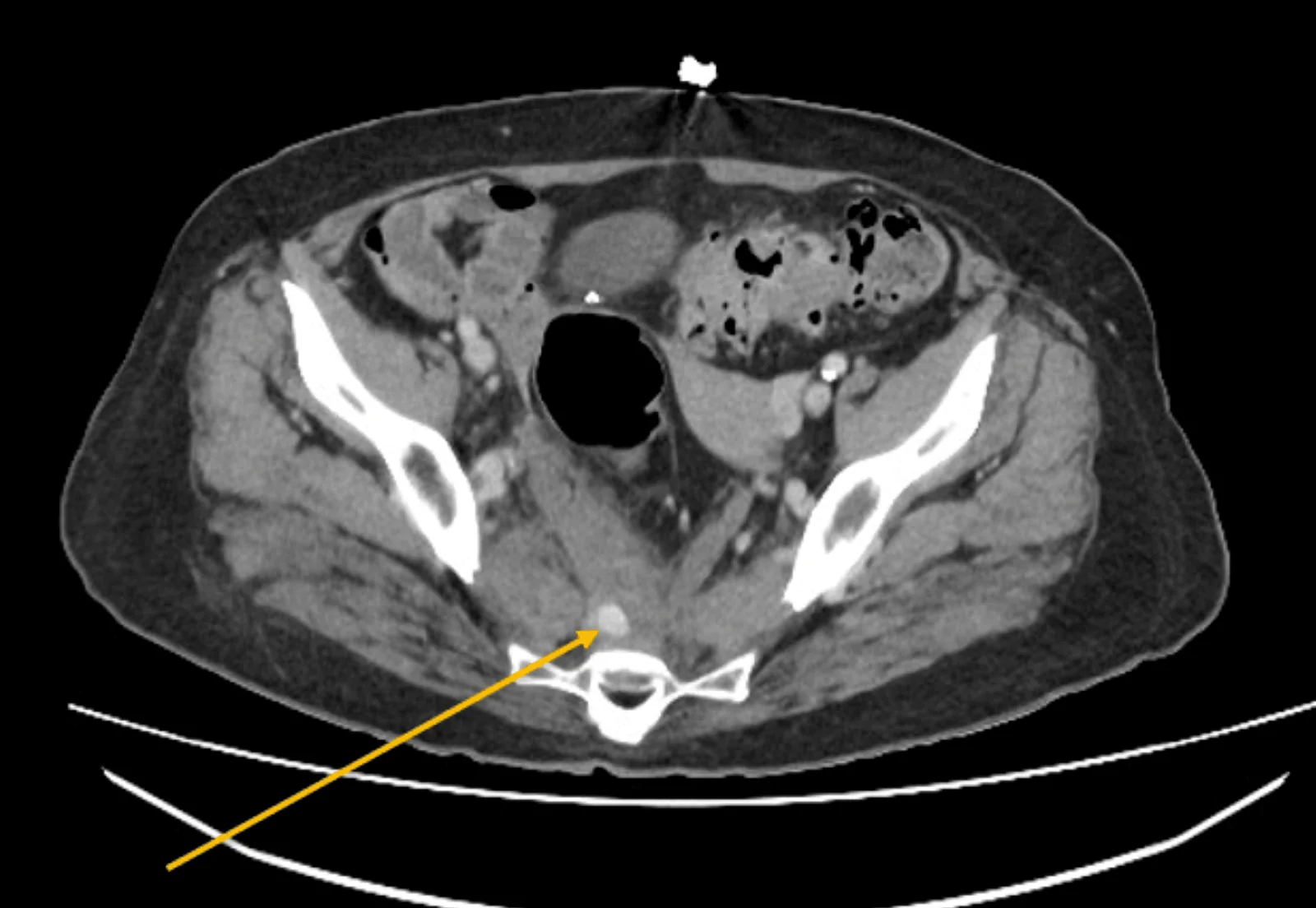
Initial contrast-enhanced axial CT of the pelvis demonstrating the focal 15 mm high-attenuation structure (yellow arrow), a possible sentinel clot or pseudoaneurysm arising from adjacent sacral or iliac vessels.

**Figure 4:**
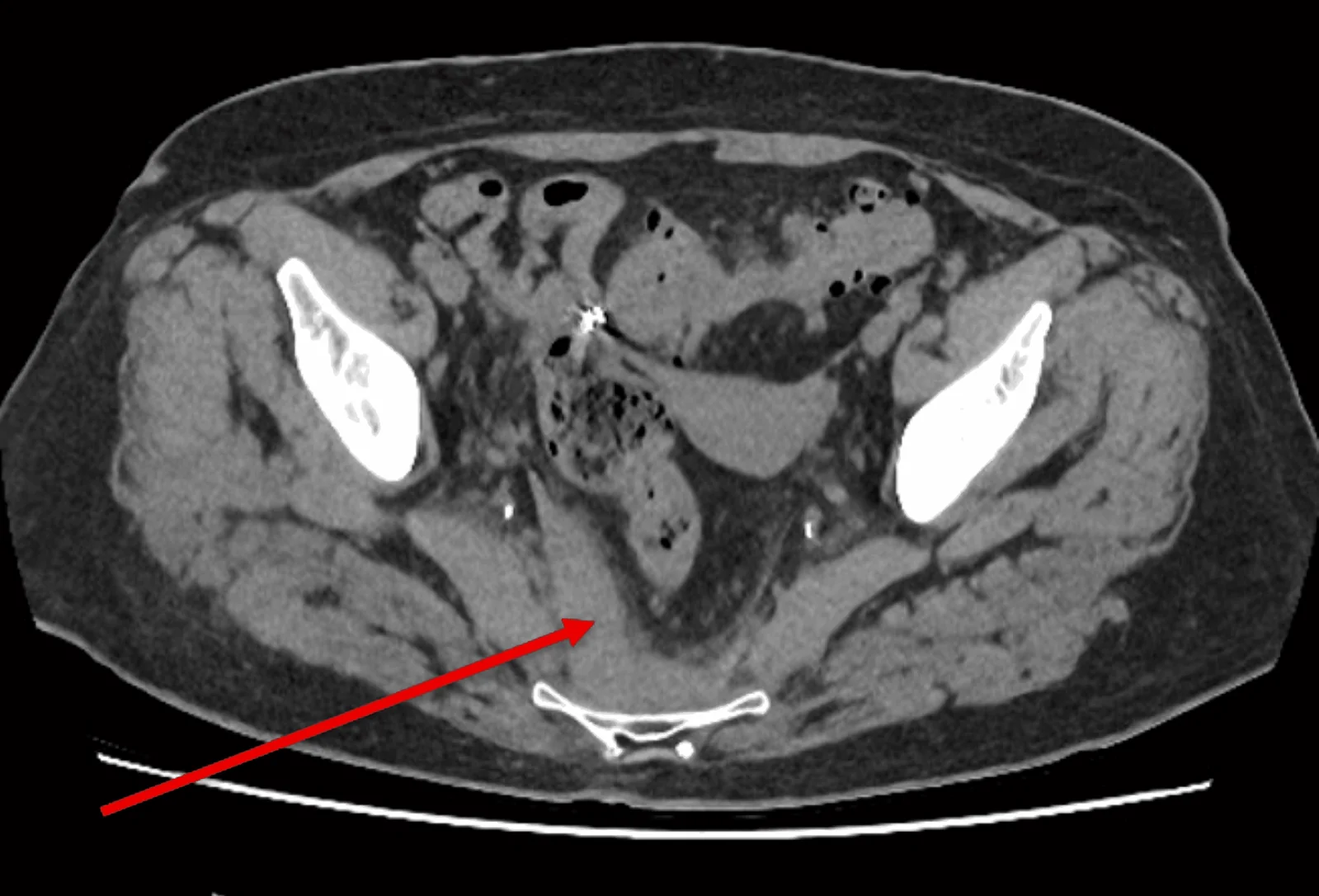
Repeat triple-phase axial CT of the pelvis showing the grossly stable right presacral haematoma (red arrow)

The patient was discharged with advice of close haematology follow-up and weekly romiplostim. At the 65-day post-discharge follow-up assessment, while receiving weekly romiplostim, her platelet count was 197 x 10^9^/L. Dose stabilisation and transition to self-administration at home are being considered.

## Discussion

ITP remains a clinical diagnosis of exclusion. In this patient, the combination of profound isolated thrombocytopenia, mucocutaneous bleeding, preserved haemoglobin and white cell count, absence of hepatosplenomegaly, negative investigations for common secondary causes, and bone marrow examination without evidence of an alternative haematological disorder supported the diagnosis of ITP [1,2]. Bone marrow examination is not routinely required in patients with otherwise typical ITP; however, it may be appropriate when clinical features are atypical, particularly in older patients or when there is an inadequate response to treatment [2].

Severe ITP is defined by the presence of bleeding symptoms at presentation sufficient to mandate treatment, or by the development of new bleeding symptoms requiring additional therapeutic intervention or an increased dose of treatment [1]. In this case, the platelet count was undetectable and was accompanied by epistaxis and subsequent petechiae. The development of an occult presacral haematoma represented a clinically severe presentation requiring urgent treatment and close monitoring.

A nationwide French study found that approximately 18% of adult ITP cases were secondary [4]. Autoimmune diseases are among the recognised conditions associated with secondary ITP [6-9]. Secondary ITP refers to ITP occurring in association with an underlying condition, either present before or identified around the time of the ITP diagnosis [7-10].

The diagnosis of secondary ITP is clearest when remission follows treatment of the underlying disorder, such as eradication of *Helicobacter pylori* infection. In other situations, the relationship is inferred from coexistence with a recognised associated disorder, such as systemic lupus erythematosus or antiphospholipid syndrome. Treatment directed at thrombocytopenia is usually similar to that used for primary ITP and commonly includes corticosteroids, with or without IVIG, according to bleeding severity. Second- and third-line treatment decisions should also take account of the nature and activity of the underlying disease [2,7-9].

The association between recently diagnosed seropositive RA and ITP in this patient raises the possibility of an autoimmune association. However, coexistence of RA and ITP does not establish a direct causal relationship, and RA-associated ITP is rare. Ichikawa et al. described five patients with concomitant RA and ITP; three responded to glucocorticoids, whereas two required IVIG or additional immunosuppressive treatment [5]. In the present case, methotrexate had not been initiated before the onset of thrombocytopenia, making methotrexate-induced marrow suppression an unlikely explanation. Platelet recovery followed romiplostim initiation after an inadequate initial response to corticosteroids and IVIG, but the contribution of delayed responses to the preceding treatments cannot be excluded.

Management of ITP is guided primarily by the severity of bleeding rather than platelet count alone. Current guidelines recommend corticosteroids as first-line treatment in adults with newly diagnosed ITP who require treatment, particularly when platelet counts are below 30 × 10⁹/L. IVIG can be added when a rapid platelet increase is required because of active bleeding or a high risk of bleeding [2,11]. Platelet transfusion is generally reserved for severe or life-threatening bleeding because transfused platelets may be rapidly cleared in ITP [2]. In the current case, profound thrombocytopenia with mucocutaneous bleeding justified urgent rescue treatment and close inpatient observation.

Thrombopoietin-receptor agonists (TPO-RAs) have an established role in persistent or chronic ITP and are increasingly considered earlier when the response to initial corticosteroid treatment is inadequate [2,11-14]. In the TRAIT study, treatment-free response was associated with earlier TPO-RA initiation after diagnosis and with secondary ITP [13]. Romiplostim stimulates thrombopoietin receptors on megakaryocyte-lineage cells, thereby increasing platelet production [12,14]. The platelet increase following the second dose in this patient was consistent with the expected onset of response within the first few weeks of treatment [12,14]. The therapeutic objective is to achieve a platelet count sufficient to maintain haemostasis and prevent clinically significant bleeding rather than to normalise the platelet count [2,11,12]. Ongoing monitoring and dose adjustment are required because excessive platelet responses and thrombotic complications may occur during TPO-RA therapy [2,12,14].

A particularly notable feature of this case was the identification of a spontaneous presacral haematoma. Presacral haematoma is an uncommon form of internal bleeding and is particularly unusual when occurring spontaneously in the absence of trauma or a recent pelvic procedure. In this patient, the haematoma was clinically occult and was identified incidentally on imaging performed during investigation of the underlying cause of the severe thrombocytopenia. Its precise time of onset and a definitive causal relationship with ITP could not be established, although profound thrombocytopenia provided a plausible bleeding tendency. The absence of symptoms did not exclude clinically relevant internal bleeding in the setting of profound thrombocytopenia.

Spontaneous atraumatic presacral haematoma has previously been reported in patients with an underlying bleeding tendency, including anticoagulant-associated bleeding [15]. The anatomical characteristics of the true pelvis may allow substantial blood accumulation, and adequate tamponade may be achieved between the rigid true pelvis and the intact covering peritoneum. Management depends on haemodynamic stability, evidence of ongoing bleeding, and the presence or absence of complications affecting adjacent structures. Conservative management may be appropriate in stable patients without active arterial bleeding, infection, or significant compression of adjacent organs, together with correction of the underlying bleeding tendency and appropriate clinical and radiological follow-up [16]. In the present case, the patient remained haemodynamically stable, and follow-up imaging demonstrated no active arterial bleeding, supporting continued conservative management.

The relationship between RA and ITP in the present case remains uncertain, and a direct causal association cannot be established. Nevertheless, the case demonstrates several clinically important features: profound thrombocytopenia with initial mucocutaneous bleeding, inadequate response to initial rescue therapy, identification of an otherwise clinically occult internal haematoma, and subsequent platelet recovery following treatment with romiplostim. It also highlights the importance of considering unusual sites of bleeding in patients with severe thrombocytopenia, even in the absence of localising symptoms.

## Conclusions

Severe ITP may occur in association with autoimmune diseases such as RA, although a direct causal relationship could not be established in this case. The clinically occult presacral haematoma highlights that significant internal bleeding may occur at unusual anatomical sites despite the absence of localising symptoms. Conservative management was appropriate in the current case because the patient remained haemodynamically stable and repeat CT demonstrated no active bleeding. Platelet recovery occurred following the initiation of romiplostim after an inadequate initial response to corticosteroids and IVIG, although delayed effects of the preceding treatments may also have contributed. Longer-term follow-up is required to assess the durability of the platelet response and whether romiplostim can be reduced or discontinued.
